# CGRPα-Expressing Sensory Neurons Respond to Stimuli that Evoke Sensations of Pain and Itch

**DOI:** 10.1371/journal.pone.0036355

**Published:** 2012-05-01

**Authors:** Eric S. McCoy, Bonnie Taylor-Blake, Mark J. Zylka

**Affiliations:** Department of Cell and Molecular Physiology, UNC Neuroscience Center, University of North Carolina at Chapel Hill, Chapel Hill, North Carolina, United States of America; Duke University, United States of America

## Abstract

Calcitonin gene-related peptide (CGRPα, encoded by *Calca*) is a classic marker of nociceptive dorsal root ganglia (DRG) neurons. Despite years of research, it is unclear what stimuli these neurons detect *in vitro* or *in vivo*. To facilitate functional studies of these neurons, we genetically targeted an axonal tracer (farnesylated enhanced green fluorescent protein; GFP) and a LoxP-stopped cell ablation construct (human diphtheria toxin receptor; DTR) to the *Calca* locus. In culture, 10–50% (depending on ligand) of all CGRPα-GFP-positive (+) neurons responded to capsaicin, mustard oil, menthol, acidic pH, ATP, and pruritogens (histamine and chloroquine), suggesting a role for peptidergic neurons in detecting noxious stimuli and itch. In contrast, few (2.2±1.3%) CGRPα-GFP^+^ neurons responded to the TRPM8-selective cooling agent icilin. In adult mice, CGRPα-GFP^+^ cell bodies were located in the DRG, spinal cord (motor neurons and dorsal horn neurons), brain and thyroid—reproducibly marking all cell types known to express *Calca*. Half of all CGRPα-GFP^+^ DRG neurons expressed TRPV1, ∼25% expressed neurofilament-200, <10% contained nonpeptidergic markers (IB4 and Prostatic acid phosphatase) and almost none (<1%) expressed TRPM8. CGRPα-GFP^+^ neurons innervated the dorsal spinal cord and innervated cutaneous and visceral tissues. This included nerve endings in the epidermis and on guard hairs. Our study provides direct evidence that CGRPα^+^ DRG neurons respond to agonists that evoke pain and itch and constitute a sensory circuit that is largely distinct from nonpeptidergic circuits and TRPM8^+^/cool temperature circuits. In future studies, it should be possible to conditionally ablate CGRPα-expressing neurons to evaluate sensory and non-sensory functions for these neurons.

## Introduction

Small-to-medium-diameter neurons in the dorsal root ganglia (DRG) have classically been divided into peptidergic and nonpeptidergic subsets [1], [2]. Many of these neurons respond to noxious thermal, mechanical and chemical stimuli, making them nociceptive, whereas others respond to innocuous stimuli, such as warming and cooling. The most widely recognized markers of peptidergic neurons are CGRP and substance P, while IB4-binding and fluoride-resistant acid phosphatase (FRAP; also known as Prostatic acid phosphatase, PAP) classically mark nonpeptidergic neurons [3], [4].

The sensory functions of these circuits were recently examined through the use of sophisticated genetic and physiological techniques. Nonpeptidergic, *Mrgprd*-expressing neurons are unmyelinated and contribute to mechanosensation but not thermosensation or cold sensation [5], [6]. Peptidergic CGRP^+^ neurons are myelinated (A-fibers) or unmyelinated (C-fibers) and, depending on fiber type, respond to nociceptive stimuli or guard hair displacement [7], [8]. TRPV1^+^ neurons, a subset of which are peptidergic [9], detect noxious thermal stimuli and some pruritogens [5], [10], [11], [12], [13]. However, the extent to which the broader class of peptidergic CGRP^+^ neurons is required for innocuous and noxious stimulus detection in mammals is currently unknown.

CGRP is not a single peptide but two separate peptides (CGRPα and CGRPβ) encoded by separate genes (*Calca* and *Calcb*). *Calca* is alternatively spliced, giving rise to CGRPα in neurons and calcitonin in thyroid C cells [14]. And, CGRPα and CGRPβ are nearly identical at the amino acid level. As a result, antibodies typically cannot distinguish CGRPα from CGRPβ, necessitating use of the term “CGRP-immunoreactivity" (CGRP-IR). CGRP-IR cells and fibers are present in multiple tissues, including the brain, stomach, intestine, skin and bladder [15], [16], [17], [18]. In studies where expression of each gene was resolved, both CGRPα and CGRPβ were expressed in the DRG although CGRPα was expressed at two-fold higher levels [16], [17].

When released peripherally from neurons, CGRPα causes vasodilatation, relaxes smooth muscle cells and contributes to migraine pathogenesis [19]. CGRPα is also released in the dorsal spinal cord and potentiates excitation caused by noxious stimuli and pronociceptive chemicals [20], [21]. CGRPα levels also regulate sensitivity to noxious heat [22]. Notably, CGRPα knockout mice have reduced behavioral responses to capsaicin and impaired heat hyperalgesia although acute heat responsiveness is not affected [23], [24], [25].

To directly study the projections and sensory functions of CGRPα neurons, we generated a knock-in mouse that expresses an axonal tracer and a conditional cell ablation construct from the *Calca*/*Cgrpα* locus. We used these mice to prospectively identify peptidergic DRG neurons in culture and show that they respond to agonists that evoke sensations of pain and itch.

## Results

### CGRPα-GFP^+^ neurons respond to agonists that evoke sensations of pain and itch

**Figure 1:**
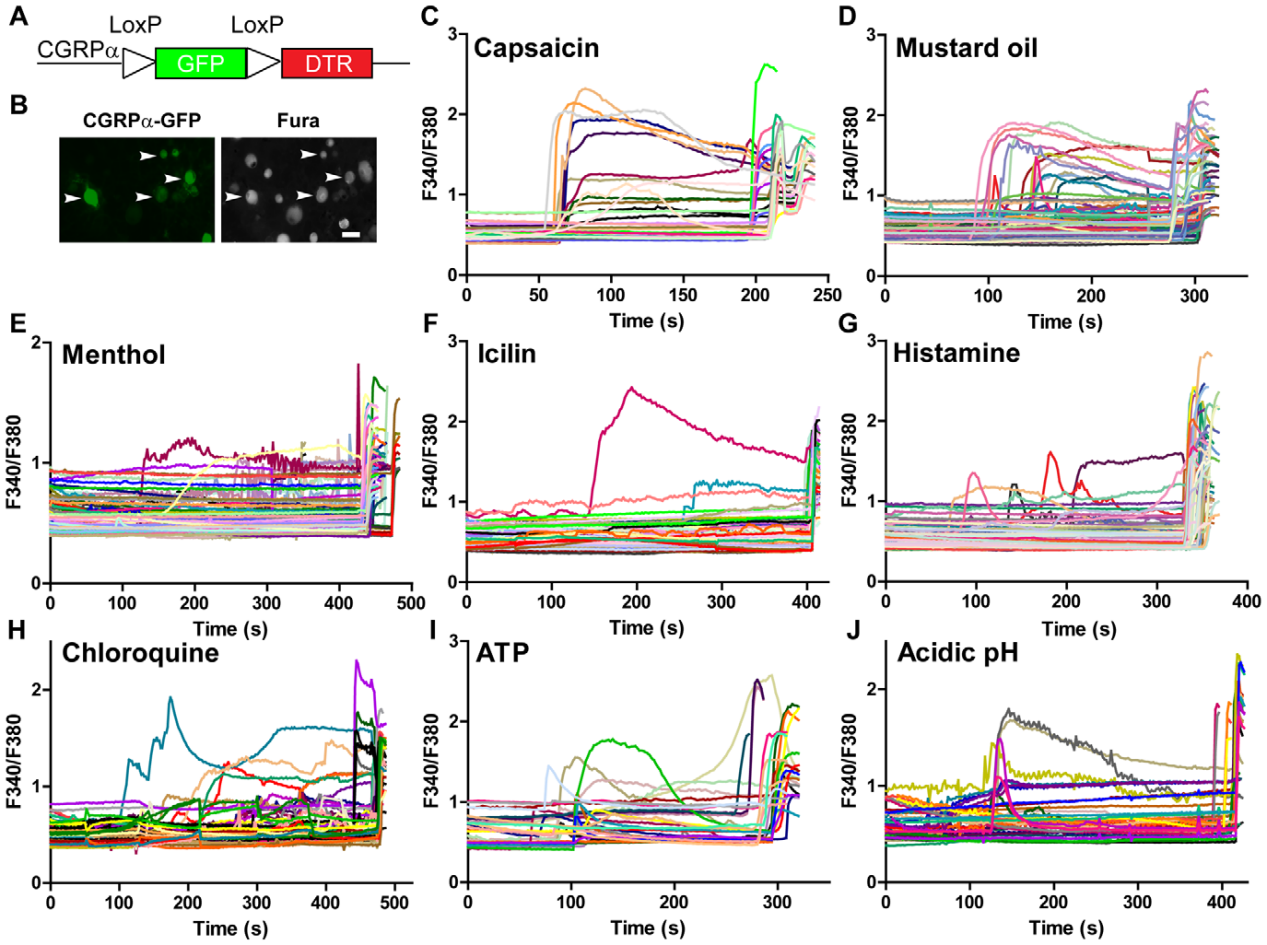
CGRPα-GFP^+^ DRG neurons respond to agonists that evoke pain and itch sensation. (A) Farnesylated GFP-DTR knocked-in to the start codon of CGRPα. Expression of DTR is conditionally blocked by LoxP flanked GFP and three polyadenylation signals (not shown). LoxP sites are oriented so that the first ATG encountered is in GFP. (B) Representative images of cultured DRG neurons from a CGRPα-GFP^+/−^ mouse after loading with Fura2-AM. Arrows point to CGRPα-GFP^+^ neurons. (C–J) Responses of CGRPα-GFP^+^ neurons from heterozygous mice to (C) capsaicin (1 µM), (D) mustard oil (100 µM), (E) menthol (200 µM), (F) icilin (4 µM), (G) histamine (100 µM), (H) chloroquine (1 mM), (I) ATP (100 µM) and (J) acidic pH, followed by stimulation with 100 mM KCl to identify healthy neurons. Scale bar in (B) right panel is 50 µm.

At the time we began this study, there were no ways to prospectively identify CGRP^+^ sensory neurons for physiological studies. To permit direct visualization of CGRP^+^ sensory neurons and axons, we knocked-in a floxed (LoxP flanked) membrane-tethered axonal tracer (farnesylated enhanced GFP) to the *Calca* locus (Fig. 1A) [26]. This floxed GFP also conditionally blocks expression of downstream DTR (Cre recombinase-dependent expression of DTR will be described in a subsequent study). Heterozygous (CGRPα-GFP^+/−^) mice, which contain one functional *Calca* allele and one GFP allele, were used throughout this study. The mice were viable and showed no obvious phenotypic or behavioral abnormalities.

We next loaded cultured DRG neurons from CGRPα-GFP^+/−^ mice with the calcium indicator Fura2-AM. CGRPα-GFP^+^ neurons were readily identifiable based on intrinsic GFP fluorescence and accounted for 39.9% of all Fura2-loaded neurons (Fig. 1B, arrowheads; n = 1292 Fura2^+^ neurons analyzed). No CGRPα-GFP^+^ cells were present from wild-type littermate controls. A majority (54.8%) of these CGRPα-GFP^+^ neurons were 17–30 µm in diameter, with the remainder being either smaller or larger.

We then monitored calcium responses in all CGRPα-GFP^+^ and CGRPα-GFP^−^ neurons to capsaicin, mustard oil, menthol, icilin, histamine, chloroquine, ATP and acidic pH (Fig. 1C–J, Table 1, Table S1, Table S2). Capsaicin is a ligand for the noxious heat- (>43°C) and acid-sensitive TRPV1 receptor [27] and activated approximately 50% of all CGRPα-GFP^+^ neurons. Mustard oil, an agonist of the irritant and noxious cold receptor TRPA1, activated 36.8±2.0% of all CGRPα-GFP^+^ neurons. Menthol, a nonselective modulator of TRPA1 and TRPM8 [28], [29], activated 14.3±5.0% of all CGRPα-GFP^+^ neurons (Table 1). A low concentration of icilin, which selectively activates TRPM8 [30], activated 2.2±1.3% of all CGRP^+^ neurons. Histamine and chloroquine evoke the sensation of itch [31] and activated ∼11% of all CGRPα-GFP^+^ neurons. The nonselective P2X and P2Y purinergic receptor agonist ATP activated 12.8±2.2% of all CGRPα-GFP^+^ neurons. Lastly, lowering the pH to between 5.0 and 6.0 activated 27.0±2.2% of all CGRPα-GFP^+^ neurons. For all ligands studied, the majority of all CGRPα-GFP^+^ responsive cells were small- to medium-diameter (Table S1). Taken together, these data indicate that peptidergic CGRPα^+^ neurons can detect diverse ligands that evoke sensations of pain and itch.

**Table 1:** Percentage of CGRPα-GFP^+/−^ DRG neurons that respond to agonists that evoke pain and itch.

Agonist	% Responders/CGRPα-GFP^+/−^	% CGRPα-GFP^+/−^Responders/Total Responders
Capsaicin	48.5±3.9	48.8±5.6
Mustard oil	36.8±2.0	32.6±8.7
Menthol	14.3±5.0	29.4±1.8
Icilin	2.2±1.3	40.0±12.2
Histamine	10.9±1.5	61.1±5.6
Chloroquine	11.6±1.1	72.7±1.8
ATP	12.8±2.2	27.3±3.4
Acidic pH	27.0±2.2	83.3±8.3

### CGRPα-GFP genetically marks a circuit that is largely distinct from nonpeptidergic and TRPM8^+^ sensory circuits

**Figure 2:**
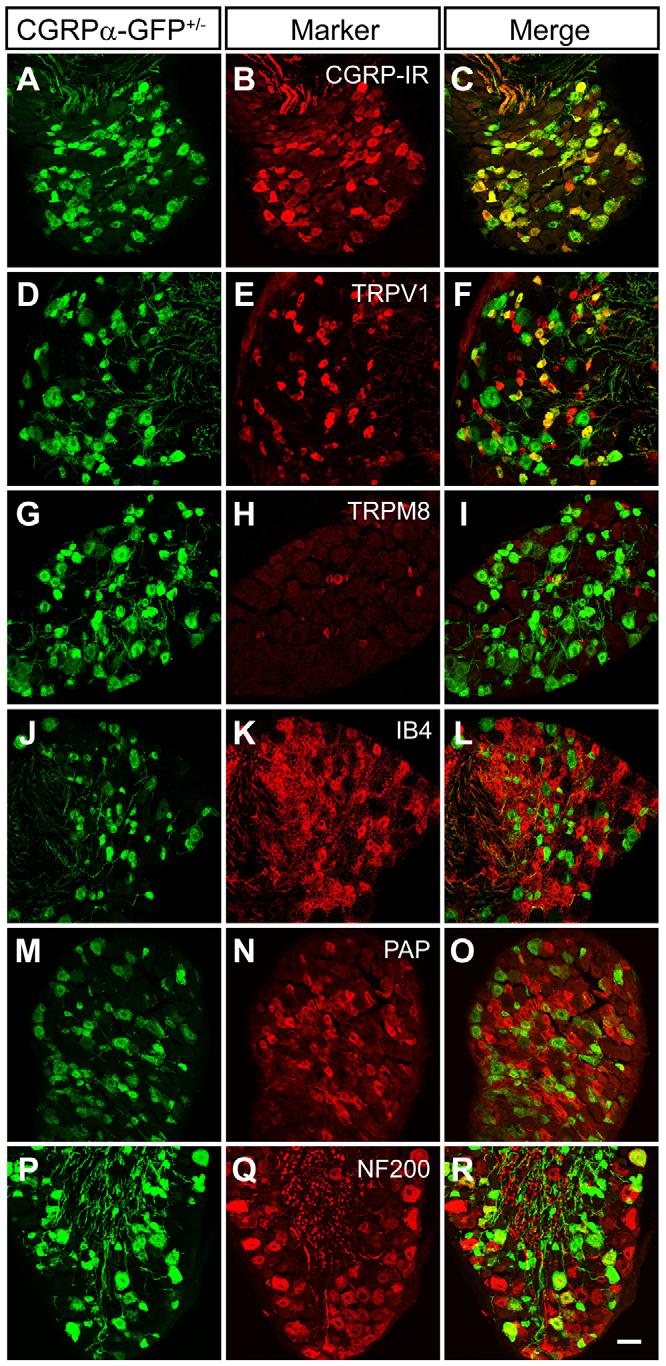
CGRPα-GFP colocalizes with peptidergic nociceptive neuron markers. Sections of L4-L6 DRG from CGRPα-GFP^+/−^ mice were stained with antibodies to GFP (A,D,G,J,M,P) and the indicated markers. Images were acquired by confocal microscopy. Scale bar in (R) is 50 µm.

To determine if CGRPα-GFP was expressed in peptidergic sensory neurons, we next immunostained sections of lumbar DRG with antibodies to GFP and various neuronal markers. We found that the vast majority (88.9±0.5%) of all CGRP-IR neurons were CGRPα-GFP^+^ (Fig. 2A–C, Table 2). Conversely, 67.8±0.8% of all CGRPα-GFP^+^ neurons were CGRP-IR. This lack of complete overlap was likely due to the greater sensitivity of GFP immunostaining—GFP filled cells in their entirety and was easier to detect than CGRP-IR, especially in cells with low levels of CGRP-IR. Interestingly, ∼10% of the CGRP-IR neurons did not colocalize with CGRPα-GFP. Because the CGRP antibody we used recognizes CGRPα and CGRPβ, these CGRP-IR-only cells could represent DRG neurons that express CGRPβ alone [16], [17]. In addition, approximately 50% of the CGRPα-GFP^+^ neurons expressed TRPV1 (Fig. 2D–F, Table 2), consistent with our functional studies above.

**Table 2:** Percentage of CGRPα-GFP^+^ neurons co-labeled with different markers.

Marker	% CGRPα-GFP^+/−^/Marker^+^	% Marker^+^/CGRPα-GFP^+/−^
CGRP	67.8±0.8	88.9±0.5
TRPV1	47.8±2.0	47.9±1.8
TRPM8	0.2±0.1	0.9±0.4
IB4	6.2±0.7	9.3±1.4
PAP	9.1±0.8	10.5±0.7
NF200	18.1±0.5	24.0±0.9
DTR	0	0

In contrast, there was little (<1%) overlap between CGRPα-GFP and TRPM8 (Fig. 2G–I), a receptor that is activated by icilin, menthol and cool temperatures [32], [33]. There was also limited overlap between CGRPα-GFP and nonpeptidergic markers (IB4 and PAP; Fig. 2J–O, Table 2), which was consistent with previous studies [3], [34]. Some (18.1±0.5%) CGRPα-GFP^+^ neurons colocalized with neurofilament-200 (NF200) (Fig. 2P–R), a marker of large-diameter neurons with myelinated axons. Importantly, DTR was not expressed in any DRG neurons (Table 2), indicating that the floxed GFP insert effectively blocks downstream transcription.

**Figure 3:**
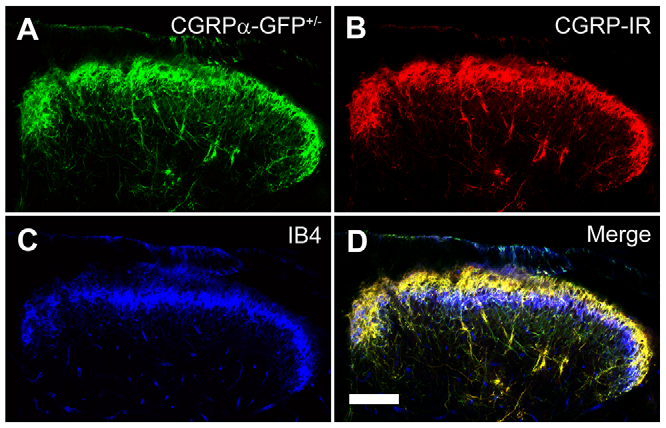
CGRPα-GFP axons terminate in dorsal spinal cord. Sections of lumbar spinal cord from CGRPα-GFP^+/−^ mice were stained with antibodies to (A) GFP and (B) CGRP. (C) IB4-binding. (D) Merged image. Images were acquired by confocal microscopy and are representative of n = 3 mice. Scale bar in (D) is 100 µm.

In the spinal cord, CGRPα-GFP (Fig. 3A) and CGRP-IR (Fig. 3B) were colocalized in lamina I and lamina II outer, with fibers extending into lamina V and towards lamina X. There was little overlap between CGRPα-GFP^+^ and IB4-binding terminals in lamina II (Fig. 3C, D), revealing segregation between peptidergic and nonpeptidergic spinal circuitry. When taken together, our data indicate that CGRPα-GFP genetically marks a distinct subset of small-to-medium- and large-diameter DRG neurons in adult mice and constitutes a circuit that is largely distinct from nonpeptidergic circuits and TRPM8^+^/cool temperature-sensing circuits.

### CGRPα-GFP is expressed in motor neurons and a small population of neurons intrinsic to the dorsal spinal cord

**Figure 4:**
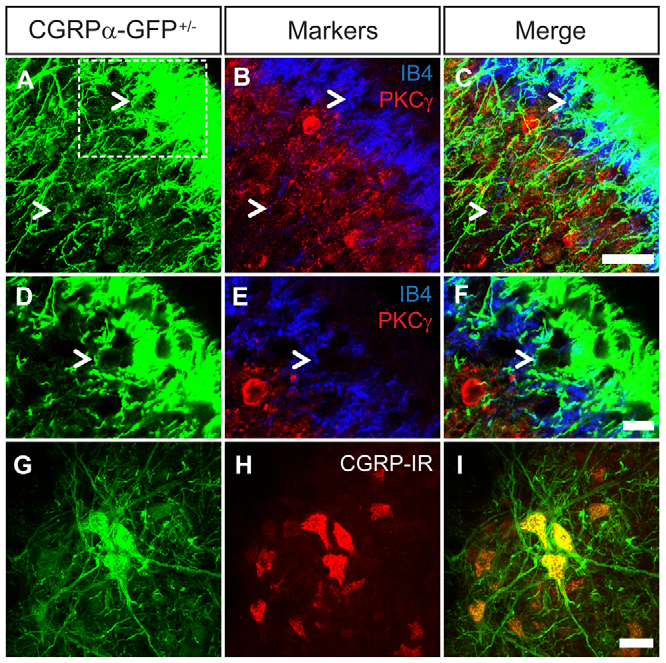
CGRPα-GFP marks a small population of neurons in lamina II/III and motor neurons in spinal cord. Sections of lumbar spinal cord from CGRPα-GFP^+/−^ mice were stained with (A–F) antibodies to GFP (green) and PKCγ (red). IB4-binding (blue). Arrowheads point to GFP^+^ cells. (D–F) Single confocal scan image from box in (A) reveals a CGRPα-GFP^+^ neuron located in lamina II. (G–I) Motor neurons stained with antibodies to GFP (green) and CGRP (red). Images were acquired by confocal microscopy and are representative of n = 3 mice. Scale bar in (C and F) is 25 µm and in (I) is 100 µm.

**Figure 5:**
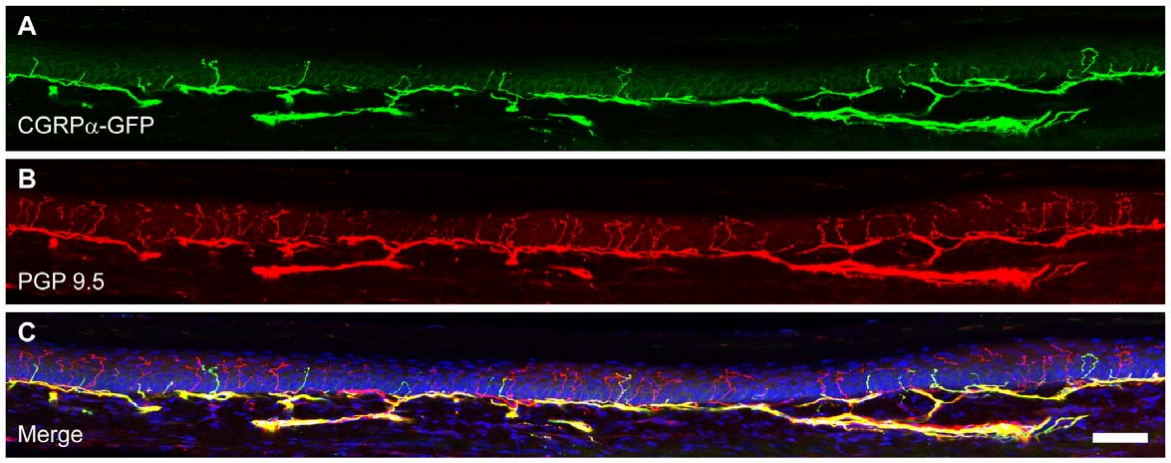
Glabrous skin montage. Sections from the glabrous skin of the hindpaw from CGRPα-GFP+/? mice were stained with antibodies to (A) GFP and (B) PGP9.5. (C) Merged images were stained with the nuclear marker DRAQ5. Images were acquired by confocal microscopy and are representative of n = 3 mice. Scale bar in (C) is 50 μm.

CGRP-IR in the dorsal horn is typically attributed to primary afferent axons and their terminals; however CGRP-IR is also present in a subset of dorsal horn neurons in rats and mice [35], [36]. To detect these cells immunohistochemically, these groups performed dorsal rhizotomies or treated animals with colchicine (colchicine arrests axonal transport, allowing CGRP to accumulate). Scattered *Cgrpα/Calca*-expressing cells were also detected in the dorsal horn by *in situ* hybridization, in Allan Brain Atlas adult spinal cord images [37]. The high sensitivity of the membrane-tethered GFP axonal tracer allowed us to detect these intrinsic CGRPα^+^ neurons without manipulating mice surgically or chemically. When examined at higher magnification, these spinal neurons were located between axon terminals of CGRPα-GFP^+^ and IB4^+^ sensory neurons, with CGRPα-GFP^+^ neurons being predominantly located in lamina II inner and lamina III (Fig. 4A–F, arrowheads). Very few of these intrinsic CGRPα-GFP^+^ dorsal horn neurons contained PKCγ (Fig. 4C,F), a marker of some lamina II and III neurons [38], [39]. In the ventral horn, CGRPα-GFP labeled many CGRP-IR motor neurons (Fig. 4G–I) along with their axons, which terminate at motor endplates in skeletal muscle (Fig. 5A–C). There were also a number of CGRP-IR motor neurons that lacked CGRPα-GFP, likely reflecting a subset of motor neurons that only express CGRPβ [40].

### CGRPα-GFP^+^ neurons innervate cutaneous and visceral tissues

**Figure 6:**
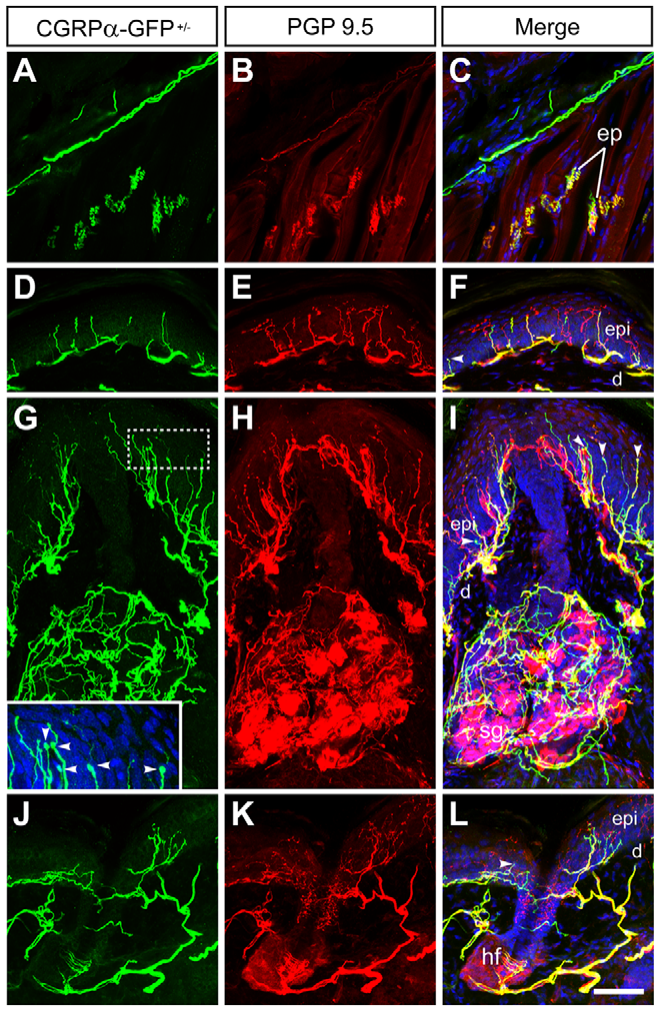
CGRP?-GFP+ axons innervate muscle and skin. Sections of hindpaw from CGRPα-GFP+/? mice were stained with antibodies to (A,D,G,J) GFP and (B,E,H,K) the pan-nerve fiber marker PGP9.5. (C,F,I,L,G-inset) Merged images were stained with the nuclear marker DRAQ5 to visualize skin cells. (AC) Nerve bundle in the subdermis and motor end plates (ep). (DF) Epidermis (epi) and upper dermis (d) from glabrous skin, (GI) sweat gland (sg) in glabrous skin and (JL) guard hair follicle (hf) in hairy skin. Arrowheads point to putative transduction spheres. Images were acquired by confocal microscopy and are representative of n = 3 mice. Scale bar in (L) is 100 μm.

CGRP-IR fibers are present in cutaneous and visceral tissues [16], [18], [26], [41], [42], [43]. Whether these fibers originate from CGRPα- and/or CGRPβ-expressing sensory neurons is unknown. To address this question, we stained a number of peripheral tissues from CGRPα-GFP^+/−^ mice with antibodies to GFP and the pan-nerve fiber marker PGP9.5. We observed CGRPα-GFP^+^ free nerve endings in the epidermis of glabrous skin (Fig. 5D–I, Fig. 6). Most of these CGRPα-GFP^+^ endings had a straight and stubby morphology that was distinct from the meandering “zig-zag" shape of PGP9.5^+^/CGRPα-GFP^−^ (presumably nonpeptidergic) fibers. We previously observed this same morphological distinction between peptidergic and nonpeptidergic fibers when targeting farnesylated GFP to *Mrgprd* ^+^/nonpeptidergic neurons [26]. Interestingly, we also noticed that some of the epidermal CGRPα-GFP^+^ fibers had small spheres at their tips (see arrowheads, Fig. 5G-inset). These spheres may simply result from membrane budding or intriguingly might constitute a novel transduction unit at the tips of some peptidergic afferents. CGRPα-GFP^+^ afferents were also present within sweat glands of glabrous skin (Fig. 5G–I). These afferents, which were also PGP9.5^+^, are likely sensory in origin because CGRPα is not expressed in sympathetic ganglia of mice [17]. In hairy skin, CGRPα-GFP^+^ fibers progressed through the dermis and terminated in the epidermis and on guard hair follicles (Fig. 5J–L).

**Figure 7:**
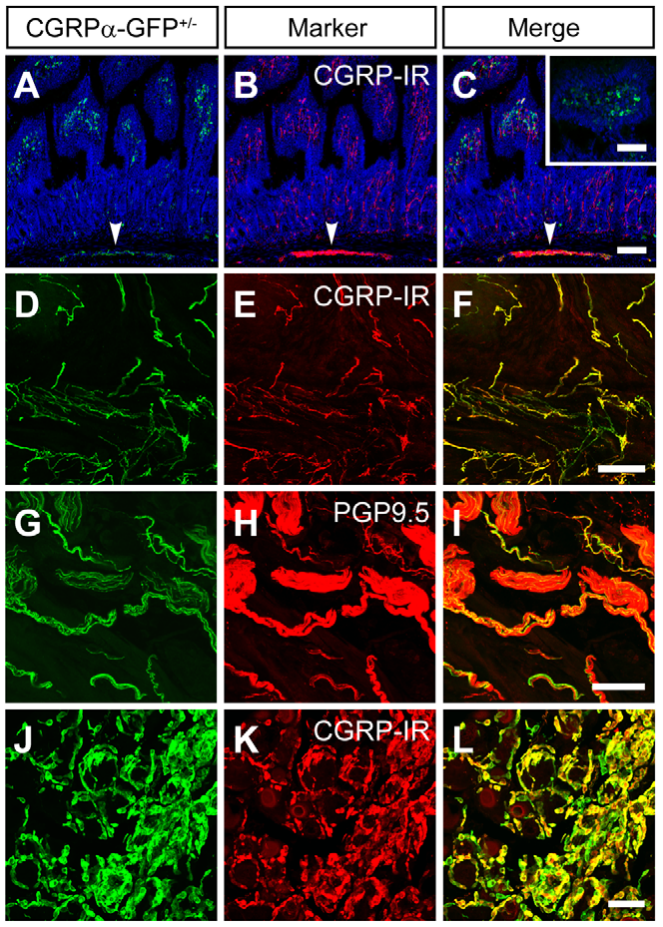
CGRPα-GFP axons and cells in visceral tissues. Sections of (A–C) small intestine, (D–I), bladder and (J–L) thyroid from CGRPα-GFP^+/−^ mice were stained with antibodies to GFP (A, D, G, J) and the indicated markers. Nuclei in (A–C) were labeled with DRAQ5. (C-inset) Section of wild-type mouse small intestine stained for GFP (green) and DRAQ5 (blue). Images were acquired by confocal microscopy and are representative of n = 3 mice. Scale bars in (C) and (C-inset) inset are 100 µm and apply to (A–C). Scale bar in (L) is 50 µm and applies to (D–L).

In addition, CGRPα-GFP^+^ fibers were present in the submucosal/smooth muscle layers of the small intestine (Fig. 7A–C), consistent with previous studies [44], [45]. There were also numerous green fluorescent cells in intestinal villi; however, these cells were not CGRPα-GFP^+^ because: a) they did not co-stain for CGRP-IR (Fig. 7B) and more importantly, b) they were detectable in wild-type mice (i.e., mice lacking GFP; Fig. 7C-inset). These cells are likely a population of autofluorescent stromal cells [46]. There were also a large number of CGRP-IR cells in the intestinal villi that were not CGRPα-GFP^+^ (Fig. 7B). These CGRP-IR-only cells likely express CGRPβ, particularly given that CGRPβ/*Calcb* is the primary CGRP gene expressed in the gut [16], [17]. Lastly, we observed CGRPα-GFP^+^ afferents in the bladder (Fig. 7D–I), a visceral tissue that is innervated by sensory afferents. When taken together, our data indicate that CGRPα-GFP^+^ neurons innervate diverse cutaneous and visceral structures.

### CGRPα-GFP labels other cell types that express *Calca*, including thyroid cells and neurons in the brain

Since GFP was targeted to exon 2 of *Calca*, an exon that is common to CGRPα and calcitonin [14], CGRPα-GFP should be present in all tissues where *Calca* is expressed. Indeed, we found that CGRPα-GFP was co-localized with CGRP-IR in parafollicular cells of the thyroid (Fig. 7J–L).

**Figure 8:**
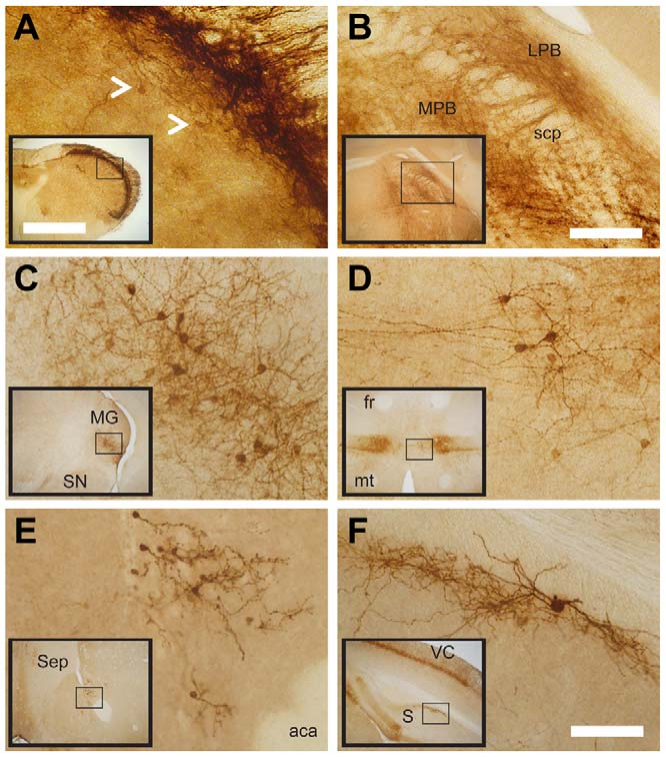
CGRPα-GFP labels neurons and axons in specific brain regions. (A–F) Brain sections from CGRPα-GFP^+/−^ mice were stained with antibodies to GFP. (A) Trigeminal spinal nucleus caudalis. Arrowheads point to labeled cells in lamina III, similar to those shown in Figure 4A–F. (B) Parabrachial nucleus. (C) Peripeduncular and posterior intralaminar thalamic nuclei. (D) Subparafascicular nucleus of the thalamus. (E) Nucleus accumbens. (F) Subiculum. Abbreviations: LPB = lateral parabrachial nucleus; MPB = medial parabrachial nucleus; scp = superior cerebral peduncle; MG = medial geniculate; SN = substantia nigra; fr = fasciculus retroflexus; mt = mammillothalamic tract; Sep = septum; aca = anterior commissure, anterior; S = subiculum; VC = visual cortex. Inset in each panel shows a lower magnification view. Scale bar in (A) inset is 1 mm and applies to all insets; (B) is 200 µm and applies to (B); (F) is 100 µm and applies to (A,C–F).

We next thoroughly mapped CGRPα-GFP expression in the brain. To do this, we immunostained adult mouse brain sections and noted all locations where CGRPα-GFP^+^ cell bodies were found (Table 3). With the exception of the abducens nucleus, Purkinje cells, cuneiform nucleus and the dorsomedial thalamic nucleus, we detected CGRPα-GFP^+^ cell bodies in all regions previously known to express CGRPα [47], [48], [49], [50]. Representative regions where cellular and/or fiber staining were observed include the spinal trigeminal nucleus caudalis (Fig. 8A), the parabrachial nucleus (Fig. 8B), the peripeduncular and posterior intralaminar thalamic nuclei (Fig. 8C), the subparafascicular nucleus of the thalamus (Fig. 8D), the nucleus accumbens (Fig. 8E), the subiculum (Fig. 8F) and weakly in the visual cortex (Fig. 8F, inset). *Calca-GFP* BAC transgenic mice produced by the GENSAT project show a similar distribution of cellular and axonal labeling in the brain [51]. Taken together, our data indicate that CGRPα-GFP knock-in mice reproducibly mark all cells and tissues that are known to express *Calca*.

**Table 3:** Cell body expression of CGRPα-GFP in different brain regions.

**Cranial Nuclei**	**-Thalamus**
Accessory facial nucleus	Anterodorsal thalamic nucleus
III Oculomotor; III Oculomotor, parvicellular	Central medial thalamic nucleus
IV, Trochlear	Gustatory thalamic nucleus
V, Trigeminal, motor	Intergeniculate leaf
V, Trigeminal, sensory nucleus	Intergeniculate thalamic nucleus
VII, Facial; perifacial zone	Mediodorsal thalamic nucleus (and
Ambiguus (IX, X motor)	subdivisions)
Nucleus of solitary tract (IX, VII, X)	Parafascicular nucleus
X, Vagus, dorsal motor	Paraventricular thalamic nucleus
XII, Hypoglossal	Peripeduncular nucleus
	Posterior thalamic nucleus
**Hindbrain/midbrain**	Posterolateral intralaminar thalamic nucleus
Cochlear nuclear complex	Subparafascicular nucleus
Inferior colliculus	Ventromedial thalamic nucleus
Inferior olive complex	Ventral posteromedial thalamic nucleus
Interpenduncular nuclei	Zona incerta
Interstitial nucleus of Cajal	
Kolliker-Fuse nucleus	**-Geniculate (medial and ventral lateral)**
Lateral lemniscus	**-Pretectal nuclei/olivary pretectal**
Nucleus of Darkschewitsch	
Parabrachial nuclei	**Telencephalon**
Periaqueductal gray	**-Cerebral cortex**
Periolivary regions	Cingulate cortex (ventral surface)
Pontine reticular nuclei	Ectorhinal complex
Raphe nuclei	Endopiriform nucleus (dorsal and ventral nuclei)
Superior colliculus	Entorhinal cortex, lateral
Tegmental nuclei	Entorhinal cortex, medial
Ventral tegmental area	Insular cortex
Vestibular nuclear complex	Motor cortex (ventral surface)
	Orbital cortex, medial and ventral
**Diencephalon**	Perirhinal cortex
**-Hypothalamus**	Piriform cortex
Anterior hypothalamic area	Retrosplenial cortex (ventral surface)
Anterior hypothalamic nuclei (posterior and	Somatosensory cortex (ventral surface)
dorsal)	Visual cortex
Arcuate nucleus	
Dorsal hypothalamic nucleus	**-Accumbens nucleus**
Dorsomedial hypothalamic nucleus (and	**-Amygdala**
subdivisions)	**-Anterior olfactory nucleus**
Lateral hypothalamic area	**-Bed nucleus of the stria terminalis, lateral and**
Lateral hypothalamus	** medial divisions**
Medial forebrain bundle	**-Caudate putamen/striatum**
Perifornical area	**-Forceps minor corpus callosum**
Periventricular hypothalamic nucleus	**-Hippocampus, subiculum**
Posterior hypothalamic area	**-Lateral septal nucleus**
Premammillary nuclei	**-Olfactory bulb**
Preoptic area	
Suprachiasmatic nucleus	
Supramammillary nucleus	
Tuberomammillary nuclei	
Ventromedial hypothalamic nucleus	

## Discussion

We generated the first knock-in reporter mouse to directly visualize and functionally study CGRPα-containing sensory neurons. While characterizing these mice, we found that CGRPα-GFP faithfully marked the peptidergic subset of DRG neurons, as well as other cell types throughout the body that express *Calca*. In contrast, cells that express *Calcb*/CGRPβ, including intramural neurons of the intestine [17], were devoid of CGRPα-GFP immunoreactivity. Our reporter mice can thus be used to discriminate *Calca*-expressing cells from cells that express *Calcb*. The membrane-tethered GFP reporter allowed us to prospectively identify live CGRPα-expressing neurons in culture for functional studies. Remarkably, half (∼50%) of all CGRPα-GFP^+^ DRG neurons expressed TRPV1 and half of all CGRPα-GFP^+^ DRG neurons responded to the TRPV1 agonist capsaicin, suggesting that CGRPα^+^ neurons may play a significant role in capsaicin and noxious thermal sensitivity *in vivo*. In addition, >50% of all histamine- and chloroquine-responsive neurons were CGRPα-GFP^+^, suggesting a major role of CGRPα-expressing neurons in histamine-dependent and histamine-independent itch. Likewise, there is a large degree (∼90%) of overlap between TRPV1/capsaicin-responsive neurons and histamine-responsive neurons [31], [52], suggesting thermal pain and histamine-dependent itch are encoded by the same class of sensory neurons.

We found little (<1%) overlap between CGRPα-GFP^+^ cells and TRPM8. And, very few (2%) CGRPα-GFP^+^ cells were activated by the cooling agent icilin (at a concentration that preferentially activates TRPM8 [30]). Likewise, others found no overlap between CGRP-IR and *Trpm8*-expression in DRG [32], [53], and no CGRP-IR neurons responded to cooling in electrophysiological studies [7]. These results collectively suggest segregation between CGRP and cool temperature-sensing/TRPM8^+^ circuits.

In contrast, Takashima *et al.* found that TRPM8-GFP and CGRP-IR overlap by ∼20% when using a BAC transgene to mark *Trpm8*-expressing neurons [54]. BAC reporters often drive higher levels of gene expression when compared to knock-in reporters, but can suffer from position effects that compromise expression specificity [51]. Thus, higher detection sensitivity and/or position effects could explain why there was a greater degree of overlap between CGRP-IR and BAC reporter driven *Trpm8* expression than we and others observed when examining endogenous *Trpm8* expression.

We also found that 14.3±5.0% of all CGRPα-GFP^+^ cells were menthol-responsive. Contrary to what is commonly stated in the literature, menthol is not a TRPM8 specific agonist. Menthol activates TRPA1 at sub- to low-micromolar concentrations and inhibits TRPA1 at higher concentrations [28], [29]. This bimodal modulation provides one of many explanations for why a smaller percentage of CGRPα-GFP^+^ neurons responded to menthol in culture than to the TRPA1 agonist mustard oil (Table 1).

With regard to position effects, it will be interesting to determine if the *Calca-GFP* BAC transgenic mouse line made by the GENSAT project reproduces CGRPα expression in DRG, brain and peripheral tissues to the same extent as our knock-in mouse [51]. In addition, it will be interesting to determine if this BAC transgenic line distinguishes *Calca*-expressing cells from *Calcb*-expressing cells. *Calca* and *Calcb* are located ∼80 kb apart in the mouse genome. This genomic proximity could contribute to their similar but not identical expression patterns. Baillie and colleagues recently used *Calca-GFP* BAC transgenic mice and optical imaging techniques to visualize an axon reflex in an individual CGRPα^+^ sensory afferent [55].

In what is perhaps the most comprehensive physiological study of CGRP^+^ sensory neurons to date, Lawson and colleagues found that CGRP-IR neurons can be classified as C-fiber and Aδ-fiber nociceptive units (responsive to noxious thermal and high threshold mechanical stimuli), unresponsive C-fibers or Aα/β guard hair afferents. None of the CGRP-IR neurons had C-cooling/cold or C-low threshold mechanoreceptive (C-LTMR) properties. These findings, combined with TRPV1 cell inactivation studies (described above) and our current work, consistently point to a role for CGRP^+^ neurons in sensing noxious heat.

CGRPα-GFP might also mark the CGRP-IR^+^ Aα/β guard hair units that were identified by Lawson and colleagues [7], particularly since CGRPα-GFP^+^ fibers terminated on guard hairs in hairy skin and ∼25% of all CGRPα-GFP^+^ neurons expressed NF200, a marker of myelinated afferents. Guard hairs add sheen to the coat of furry mammals, are often water repellent, and drive activity in sensory afferents when deflected [7], [56], [57]. Whether activation of guard hair afferents has sensory and/or non-sensory functions in mammals is currently unknown.

Ultimately, it should be possible to directly evaluate the *in vivo* functions of CGRPα^+^ sensory neurons by taking advantage of the LoxP-stopped DTR that we knocked-in immediately behind GFP (Fig. 1A). DTR, when combined with injections of diphtheria toxin, can be used to conditionally ablate cells and neurons in adult mice [5], [58]. Importantly, DTR expression was completely blocked in DRG (Table 2). We engineered DTR so that its ATG start codon will precisely substitute for the start codon of GFP upon CRE recombinase-mediated excision. DTR should thus be expressed in all cell types that jointly express CGRPα and CRE recombinase (including cells that expressed CRE at any time during development). When crossed with sensory neuron selective lines, such as *Nav1.8-Cre* or *Advillin-Cre* [59], [60], [61], [62], this could permit selective expression of DTR in DRG neurons while maintaining GFP expression in all other *Calca*-expressing cell types. Given that *Calca* is expressed in many other cell types, this strategy could be broadly employed to genetically label, ablate and study the function of diverse peptidergic CGRPα-containing cell types throughout the brain and body.

## Materials and Methods

All procedures and behavioral techniques involving vertebrate animals were approved by the Animal Care and Use Committee at the University of North Carolina at Chapel Hill.

### Molecular Biology

Recombineering was used to generate *Calca* targeting arms from a C57BL/6-derived bacterial artificial chromosome (BAC; RP24-136021). The start codon located in exon 2 is common to CGRPα and calcitonin and was replaced with an *AscI* site to facilitate cloning of an axonal tracer and a conditional cell ablation construct: *AscI*-LoxP-EGFPf-3x pA-LoxP-DTR-pA-Frt-PGK-NeoR-Frt-*AscI*. EGFPf = farnesylated enhanced GFP [26]. DTR = human diphtheria toxin receptor [58]. NeoR = neomycin resistance. The LoxP sites were oriented so that the first ATG encountered was in GFP or, after Cre recombinase-mediated excision, DTR. Correct targeting was confirmed in 5.8% of all embryonic stem cell clones by Southern blotting using flanking 5′ and 3′ probes and a NeoR internal probe. High percentage chimeras were crossed to C57BL/6 females to establish germline transmission and then crossed to ACTFLPe mice (B6.Cg-Tg(ACTFLPe)9205Dym/J, Jackson Laboratory) to remove the Frt-flanked selection cassette (removal confirmed by PCR). Next, mice were backcrossed to C57BL/6 to remove the ACTFLPe allele (removal confirmed by PCR) and then backcrossed to C57BL/6 mice for 8 generations to establish the CGRPα-GFP knock-in line. As a technical note, we were only able to detect GFP expression in DRG neurons after removal of the PGK-NeoR selection cassette.

### Calcium Imaging

Adult (4–6 week old) male CGRPα-GFP^+/−^ mice were decapitated, DRG were dissected then neurons were dissociated using collagenase (1 mg/mL; Worthington, CLS1) and dispase (5 mg/mL; Gibco, 17105-041) in DH10 media (1∶1 Ham's DMEM/F12, 10% FBS and 1% penicillin/streptomycin) [63], [64]. Medium was supplemented with 25 ng/mL of glial-derived neurotrophic factor (GDNF; Upstate, GF030). The neurons were plated onto coverslips coated with 0.1 mg/mL poly-D-lysine and 5 µg/mL laminin. After 24 h, neurons were washed 2× with Hank's balanced salt solution (HBSS) and incubated for 1 h with 2 µM Fura2-AM in the dark at room temperature. Next, the cells were washed 3× with HBSS and maintained at room temperature for 30 min prior to imaging. After a 60 s baseline, agonists (1 µM capsaicin, 100 µM mustard oil, 200 µM menthol, 4 µM icilin, 100 µM ATP, 100 µM histamine, 1 mM chloroquine or acidic pH 5–6 HBSS) were perfused onto the neurons. Following activation, cells were perfused with HBSS to remove the agonist, which was followed by addition of 100 mM KCl to determine the total number of neurons present. Images were acquired on a Nikon Eclipse Ti microscope (Nikon, Melville, NY). GFP^+^ neurons were identified by eye, and then a 500 ms exposure was used to image the cells. CGRPα-GFP^+^ neurons of all sizes were included in the analysis. Following addition of agonist, only neurons with responses greater than 15% of baseline were scored as responders.

### Histology

Mice were sacrificed by overdosing with pentobarbital. The thyroid, brain, bladder, hindpaw skin, lumbar DRG, lumbar spinal cord and small intestine were dissected and immersion-fixed in 4% paraformaldehyde (5 h, 24 h, 5 h, 3 h, 4 h, 8 h and 2 h, respectively) and were cryopreserved in 30% sucrose at 4°C. Tissue was embedded in TissueTek and cryosectioned (20 µm for small intestine and DRG; 40 µm for thyroid, bladder and spinal cord; 50 µm for brain and skin). Sections were either immunostained free-floating or thaw mounted onto SuperFrost Plus slides and stored at −20°C until needed.

Tissue was rehydrated in PBS, rinsed with TBST (0.05 M Tris, 2.7% NaCl, 0.3% Triton-X 100, pH 7.6), then blocked with 10% neat donkey serum (NDS) in TBST for 1 h at room temperature. Sections were incubated overnight at 4°C with primary antibodies. The following reagents were used: Isolectin *Griffonia simplicifolia* IB4, Alexa 568 conjugate (1∶100, Invitrogen, I21412), chicken anti-GFP (1∶600; Aves Labs, GFP-1020), rabbit anti-GFP (1∶600; Invitrogen, A11122), rabbit anti-CGRP (1∶750; Peninsula, T-4032), sheep anti-CGRP (1∶250; Enzo Life Sciences, CA1137), mouse anti-NeuN (1∶200; Millipore, MAB377), chicken anti-PAP (1∶4,000; Aves Labs), rat anti-TRPM8 (1∶100; a generous gift from Masatoshi Takeichi) [65], rabbit anti-PGP9.5 (1∶500; Ultraclone), rabbit anti-NF200 (1∶500; Sigma, N4142) and goat anti-HB-EGF (1∶1,000; R&D Systems, AF-259-NA), which labels DTR. Tissue was rinsed and then blocked for 1 h in 10% NDS in TBST. Sections were incubated for 2 h in 10% NDS in TBST with Alexa fluor-conjugated secondary antibodies (Invitrogen). DRAQ5 (1∶10; Cell Signaling, 4084) was used to label nuclei. PAP, TRPV1 and DTR immunostaining was performed using amplification, as described previously [66]. All fluorescent images were obtained using a Zeiss LSM 510 confocal microscope (Zeiss, Thornwood, NY).

For diaminobenzidine (DAB) staining, brain sections were processed as described above with chicken anti-GFP (1∶5,000). On day 2, the sections were washed in TBST and then blocked for 30 min. Sections were incubated for 2 h in biotinylated donkey anti-chicken IgG (1∶500), which was followed by washes in TBST. Sections were incubated with the Vectastain ABC complex in TBST for 2 h and washed. Sections were treated with a DAB solution (0.02% DAB, 0.01% H_2_O_2_ and 0.005% nickel ammonium sulfate in TBST) for 15 min. Following TBST washes and a PBS rinse, the sections were immersed in 0.2% gelatin in water, mounted onto Superfrost Plus slides and then air-dried for 4 days. Lastly, the sections were dehydrated with graded ethanols, cleared with xylene and coverslipped with DPX.

## Supporting Information

Table S1 **Percentage of CGRPα-GFP+/− DRG neurons of a given size class (small, medium, large diameter) that respond to the indicated agonists.**
(DOCX)

Table S2 **Percentage of GFP-negative DRG neurons of a given size class (small, medium, large diameter) that respond to the indicated agonists.**
(DOCX)
