# Supplementary material for: CGRPα-Expressing Sensory Neurons Respond to Stimuli that Evoke Sensations of Pain and Itch
Source: PLoS One. 2012 May 1;7(5):e36355. doi: 10.1371/journal.pone.0036355 (PMC3341357; doi:10.1371/journal.pone.0036355)
Supplement: Table S1 — Percentage of CGRPα-GFP+/− DRG neurons of a given size class (small, medium, large diameter) that respond to the indicated agonists. (DOCX) [file pone.0036355.s001.docx]

**Table S1.** Percentage of CGRPα-GFP^+/-^ DRG neurons of a given size class (small, medium, large diameter) that respond to the indicated agonists.

|  | **% Responders/** | **% Responders/** | **% Responders/** |
| --- | --- | --- | --- |
| **Agonist** | **CGRPα-GFP^+/-^** | **CGRPα-GFP^+/-^** | **CGRPα-GFP^+/-^** |
|  | **<17 μm** | **17-30 μm** | **>30 μm** |
| Capsaicin | 12.1 ± 5.5 | 36.4 ± 7.5 | 0 |
| Mustard Oil | 13.2 ± 4.1 | 23.7 ± 0.1 | 0 |
| Menthol | 8.6 ± 4.2 | 5.7 ± 0.8 | 0 |
| Icilin | 1.1 ± 0.9 | 1.1 ± 1.3 | 0 |
| Histamine | 3.1 ± 0.2 | 6.3 ± 2.7 | 1.6 ± 1.5 |
| Chloroquine | 7.2 ± 2.2 | 4.3 ± 2.8 | 0 |
| ATP | 7.7 ± 4.8 | 5.1 ± 2.5 | 0 |
| Acid | 8.1 ± 3.4 | 16.2 ± 4.1 | 2.7 ± 2.9 |
