# Supplementary material for: CGRPα-Expressing Sensory Neurons Respond to Stimuli that Evoke Sensations of Pain and Itch
Source: PLoS One. 2012 May 1;7(5):e36355. doi: 10.1371/journal.pone.0036355 (PMC3341357; doi:10.1371/journal.pone.0036355)
Supplement: Table S2 — Percentage of GFP-negative DRG neurons of a given size class (small, medium, large diameter) that respond to the indicated agonists. (DOCX) [file pone.0036355.s002.docx]

**Table S2.** Percentage of GFP-negative DRG neurons of a given size class (small, medium, large diameter) that respond to the indicated agonists.

|  | **% Responders/** | **% Responders/** | **% Responders/** |
| --- | --- | --- | --- |
| **Agonist** | **GFP^-^ Cells** | **GFP^-^ Cells** | **GFP^-^ Cells** |
|  | **<17 μm** | **17-30 μm** | **>30 μm** |
| Capsaicin | 13.8 ± 7.5 | 28.7 ± 0.7 | 0 |
| Mustard Oil | 7.0 ± 1.4 | 40.4 ± 4.8 | 1.8 ± 1.2 |
| Menthol | 7.5 ± 5.3 | 3.8 ± 1.6 | 0 |
| Icilin | 4.1 ± 5.0 | 0 | 0 |
| Histamine | 5.4 ± 3.7 | 8.1 ± 0.5 | 0 |
| Chloroquine | 1.7 ± 3.7 | 3.4 ± 3.8 | 0 |
| ATP | 5.5 ± 0.3 | 16.4 ± 1.1 | 1.8 ± 1.1 |
| Acid | 2.3 ± 1.9 | 2.3 ± 1.9 | 0 |
